# Screen Time, Fatigue, Obesity and Physical Inactivity: Health Correlates of Problematic Smartphone Use in Adolescents

**DOI:** 10.1111/cch.70237

**Published:** 2026-01-19

**Authors:** Mohamed Yaakoubi, Ahmed Ghorbel, Hiba Abdelkafi, Liwa Masmoudi, Adnene Gharbi, Omar Trabelsi

**Affiliations:** ^1^ High Institute of Sports and Physical Education of Kef University of Jendouba Kef Tunisia; ^2^ Research Unit: Physical Activity, Sport and Health, UR18JS01, at the National Observatory of Sport Tunis Tunisia; ^3^ The High Institute of Applied Studies in Humanities of Mahdia, Department of Education Sciences University of Monastir Mahdia Tunisia; ^4^ High Institute of Sports and Physical Education of Sfax University of Sfax Sfax Tunisia

**Keywords:** adolescence, fatigue, gender differences, overweight, physical activity, sedentary behaviour, smartphone addiction

## Abstract

**Objective:**

This study aimed to determine the prevalence of problematic smartphone use (PSU) among Tunisian adolescents, examine variations by sex and age and investigate associations with physical activity, fatigue and obesity while assessing the potential protective role of sports participation.

**Methods:**

A cross‐sectional study of 960 adolescents (53% female), aged 14–16 years utilized validated Arabic instruments including the Smartphone Addiction Scale—Short Version (SAS‐SV), International Physical Activity Questionnaire (IPAQ‐SF) and Fatigue Assessment Scale (FAS). Anthropometric measurements followed WHO protocols, and objective screen time data were collected from device tracking features. Statistical analyses included Mann–Whitney *U* tests, chi‐square tests, Spearman's correlations and logistic regression models with sex stratification, all adjusted for multiple comparisons using Benjamini–Hochberg correction.

**Results:**

PSU prevalence was 15.7%, with significantly higher rates in males (*p* < 0.001) and during mid‐adolescence (*p* < 0.001). Adolescents with PSU showed substantially higher screen time (435 vs. 155 min/day, *p* < 0.001), reduced vigorous physical activity (0 vs. 480 MET‐min/week, *p* < 0.001), increased obesity (12.6% vs. 0.7%, *p* < 0.001) and more severe fatigue (50.3% vs. 5.8%, *p* < 0.001). Sports participation demonstrated protective effects against PSU (OR = 0.4–0.6), with significantly stronger effects in females (interaction *p* = 0.03).

**Conclusion:**

PSU represents a significant public health concern among Tunisian adolescents, with a prevalence of 15.7% that is higher in males and mid‐adolescents. It is associated with excessive screen time, inactivity, elevated BMI and fatigue, whereas sports participation offers a protective effect, particularly among girls.

## Summary

- Problematic smartphone use (PSU) affects one in six adolescents and clusters with physical inactivity, fatigue and higher BMI, making it a relevant public health issue for schools and primary care.
- PSU is closely linked to excessive screen time and low physical activity, supporting prevention approaches that promote active alternatives rather than screen restriction alone.
- Sports participation is a clear protective factor against PSU, obesity and severe fatigue, indicating its value as a practical and scalable intervention.
- Sex differences are clinically and policy relevant, with stronger risk associations in males and stronger protective effects of sports in females.
- Longitudinal and intervention studies are needed to clarify causal pathways and guide effective prevention strategies using objective screen‐time measures.

## 1 Introduction

The rapid expansion of smartphone technology in developing countries has transformed social interaction, learning and entertainment. At the same time, it has raised pressing public health concerns, particularly regarding problematic smartphone use (PSU). PSU refers to compulsive, uncontrolled engagement with smartphones that interferes with daily functioning, mirroring behavioural patterns observed in other addictive conditions (Billieux 2012; M. D. Griffiths and Kuss 2017). Adolescents are especially vulnerable to PSU due to neurodevelopmental immaturity, heightened peer sensitivity and strong reward‐seeking tendencies (Casey et al. 2008). Understanding its prevalence and health correlates in this age group is crucial for informing adolescent health policy and preventive strategies.

Global evidence suggests that PSU is widespread, though estimates vary considerably. A systematic review estimated a pooled prevalence of 37.1% (Lu et al. 2024). For example, Lee et al. (2023) reported a 25.1% prevalence among South Korean adolescents, whereas Tunisian studies documented far higher estimates, ranging from 16% in community samples to nearly 69% among students (Turki et al. 2023). Such variation reflects the influence of technological environments and sociocultural factors. Tunisia represents a particularly compelling context, with smartphone ownership reaching 85% of the population (Reppas and Muschert 2024). Despite this rapid digital expansion, robust epidemiological data on PSU among Tunisian adolescents remain scarce.

Comparable heterogeneity is found in other regions. Tomczyk et al. (2024) observed that 17.2% of adolescents in Bosnia and Herzegovina displayed high‐intensity PSU, whereas Chao et al. (2022) reported 11.8% in Taiwanese students, and Alwazzeh et al. (2024) found 67.8% among Syrian undergraduates. These differences highlight both cultural and infrastructural influences as well as inconsistencies in measurement approaches.

PSU carries significant health consequences. These are often explained by the displacement hypothesis, which posits that time spent on screens directly replaces time available for physical activity and other health‐promoting behaviours, leading to a more sedentary lifestyle (Ng et al. 2023). Longitudinal analyses indicate that fatigue plays a central role in linking PSU with depressive symptoms as well as broader health problems (Wang et al. 2025). Proposed mechanisms include sleep disruption related to blue light exposure, cognitive overload and reduced restorative activities. Consistent with this pathway, empirical evidence supports this pathway: Adolescents with high PSU report poorer sleep quality, greater fatigue and lower self‐perceived health (Chao et al. 2022). Such findings underscore fatigue as a key consequence that mediates the adverse effects of PSU on both physical and mental health.

Physical inactivity and metabolic risk have also been consistently associated with PSU. Li et al. (2023) demonstrated through a systematic review of randomized trials that exercise interventions significantly reduced smartphone addiction, particularly when programs involved mixed‐skill activities lasting 30–60 min per session. Similarly, Zhang (2024) found robust negative associations between physical activity and PSU across cultural contexts. The metabolic implications are notable: Byun et al. (2024) reported that adolescents using smartphones for more than 3 h daily were nearly three times more likely to be obese compared to peers with less than 1 h of use, although each additional hour of screen exposure increased overweight risk by 8% (Mayarestya et al. 2021). These findings highlight PSU as both a behavioural and metabolic risk factor.

Sports participation may offer a protective buffer. Structured exercise programmes not only reduce PSU (Li et al. 2023) but also provide routines that limit screen exposure. Furthermore, from the perspective of self‐determination theory, sports can fulfil core psychological needs for competence, autonomy and relatedness, potentially reducing the reliance on smartphones for need satisfaction (Deci and Ryan 2008). Evidence from Spain confirms that adolescents engaged in sports report healthier diets and greater psychological well‐being, whereas technology overuse predicts poorer outcomes (Mateo‐Orcajada et al. 2023). Such findings suggest that sports participation could mitigate the negative health correlates of PSU.

Despite growing international evidence, critical gaps persist in Tunisia and similar settings. Most studies rely on self‐reported estimates, which underestimate actual smartphone use compared with digital tracking (Brosnan et al. 2025; Wade et al. 2021). Conceptual inconsistencies also remain, as divergent scales and definitions of PSU complicate cross‐study comparisons (Abendroth et al. 2020; M. Griffiths 2005). Furthermore, North African research rarely integrates contextual factors such as physical activity, rest time or sports participation (Sfendla et al. 2018). Addressing these gaps is essential to developing culturally appropriate prevention strategies.

The present study aims to determine the prevalence of PSU among Tunisian adolescents aged 14–16 years, examine its variation across sex and age and assess associations with objectively measured screen time, physical activity, fatigue and obesity. Beyond estimating prevalence, we investigated the role of overall screen time in shaping health outcomes and tested whether sports participation moderates these associations, examining its potential as a protective factor grounded in behavioural displacement and need‐satisfaction frameworks. Collectively, this work seeks to clarify how patterns of screen engagement contribute to adolescent health in Tunisia, where robust epidemiological evidence remains limited despite rapid digital expansion.

## 2 Methods

### 2.1 Study Design and Participants

This cross‐sectional study was conducted between March and June 2023 in three urban public middle schools in Tunisia using a convenience sampling approach during routine school health assessments. The study protocol was reviewed and approved by the Regional Ethics Committee (Protocol No. 0554/2023). Written informed consent and adolescent assent were obtained.

A total of 1015 adolescents were initially eligible. Exclusion criteria were applied to enhance internal validity: (1) Adolescents with diagnosed neurodevelopmental disorders, physical disabilities, chronic medical conditions, recent injuries or relevant medications were excluded, as these conditions could independently affect physical activity, fatigue and digital media use patterns, creating potential confounding pathways (Liu et al. 2024; Patel and Greydanus 2008; Russo 2022); (2) to mitigate confounding from disordered eating, participants scoring ≥ 2 on the Sick, Control, One, Fat, Food (SCOFF) questionnaire (Aoun et al. 2015; Rosen and Committee on Adolescence 2010) were excluded; (3) to ensure data accuracy, 55 participants were excluded because their devices lacked screen‐time tracking (iOS Screen Time/Android Digital Wellbeing), were shared, or used restrictive modes (‘Downtime’, ‘Focus’) during the recording week (Ellis et al. 2019).

The final analytical sample therefore consisted of 960 adolescents (53% female), aged 14–16 years. PSU was classified using gender‐specific SAS‐SV cut‐offs (≥ 31 for males, ≥ 33 for females).

### 2.2 Sample Size and Power

An a priori power analysis was conducted using G*Power Version 3.1.9.7 (Faul et al. 2007). For binary logistic regression, a minimum sample size of 210 participants was required to detect an odds ratio of 1.8, corresponding to a medium effect size based on G*Power's recommended approximation for logistic regression models (*f* ^2^ ≈ 0.15), assuming an expected event rate (PSU prevalence) of 0.20, α = 0.05 and statistical power of 0.95. The expected event rate was informed by preliminary data and regional epidemiological studies. The final analytical sample (*N* = 960) therefore provided adequate power to detect smaller effect sizes.

### 2.3 Data Collection and Measures

Data were collected through structured, face‐to‐face interviews conducted by six trained interviewers using tablets. Training emphasized standardized protocols, cultural sensitivity and real‐time cross‐verification of self‐reported data against device‐tracked screen time. Pilot testing (*n* = 30) confirmed feasibility. Inter‐rater reliability was high (κ > 0.85), and quality control included duplicate entry verification and daily data audits. The following validated instruments and procedures were used.

### 2.4 Anthropometric Measures

Height and weight measurements were sourced from existing school health records, where trained nurses collected data using standardized equipment. Body mass index (BMI) was calculated as weight/height^2^ (kg/m^2^). Age‐ and sex‐adjusted BMI z‐scores were derived using WHO Anthro‐Plus software (v1.0.4) based on WHO Growth References (5–19 years).

Participants were classified into four weight categories: underweight (z‐score < −2 SD), normal weight (z‐score ≥ − 2 to ≤ + 1 SD), overweight (z‐score > + 1 SD) and obesity (z‐score > + 2 SD) (Weir and Jan 2019).

### 2.5 Measures

#### 2.5.1 PSU

Assessed using the 10‐item Smartphone Addiction Scale—Short Version (SAS‐SV; Kwon et al. 2013). The Arabic version demonstrated excellent reliability; Cronbach's α = 0.89 (Sfendla et al. 2018). Sex‐specific cut‐offs were applied (≥ 31 for males, ≥ 33 for females).

#### 2.5.2 Screen Time

Objectively measured via built‐in device tracking (iOS Screen Time/Android Digital Wellbeing), cross‐verified during interviews (Ellis et al. 2019).

#### 2.5.3 Physical Activity

Measured using the Arabic short form of the International Physical Activity Questionnaire (IPAQ‐SF) (Awadalla et al. 2014); Cronbach's α = 0.83. Participants were classified into low (< 600 MET‐min/week), moderate (600–3000 MET‐min/week) or high (> 3000 MET‐min/week) activity levels per WHO guidelines.

#### 2.5.4 Fatigue

Assessed with the 10‐item Fatigue Assessment Scale (FAS); Arabic version α = 0.88–0.91 (Alhanbali et al. 2023). Scores were categorized as no fatigue (< 22), mild/moderate (22–34) or severe (≥ 35).

#### 2.5.5 Sports Participation

Defined as cumulative years of structured, coach‐supervised training and competition, assessed via self‐report validated against school records (Jin et al. 2021).

### 2.6 Statistical Analysis

Data were analysed using SPSS Version 29. Continuous variables were expressed as medians with interquartile ranges (IQR) after normality assumptions were violated (Shapiro–Wilk *p* < 0.05). Group comparisons utilized Mann–Whitney *U* tests for continuous variables and chi‐square tests for categorical variables, with effect sizes reported as *r* = |Z|/√N and Cramer's V, respectively.

Bivariate relationships were assessed using Spearman's correlation coefficients (ρ). Separate binary logistic regression models were constructed for SAS‐SV scores and screen time due to multicollinearity (VIF > 5.0), with all models adjusted for age, sex and BMI. For all logistic regression models, reference categories were defined as the absence of the condition under investigation (e.g., NSU) and no prior sports participation (0 years). Sex‐stratified analyses were conducted to explore potential gender‐specific associations, with female sex specified as the reference category in stratified models.

Results are presented as adjusted odds ratios (aOR) with 95% confidence intervals. The Benjamini–Hochberg procedure controlled for false discovery rate across multiple comparisons. Regression assumptions were verified through residual analysis and VIF diagnostics.

## 3 Results

### 3.1 Participant Characteristics by Smartphone Use Status

The final analytical sample comprised 960 adolescents, of whom 15.7% (*n* = 151) met the criteria for PSU. As shown in Table 1, adolescents with PSU were statistically significantly older, reported substantially greater daily screen time and higher Smartphone Addiction Scale—Short Version scores and exhibited a statistically significantly different BMI distribution compared with NSUs (all *p* < 0.001). Notably, the PSU group showed markedly higher proportions of overweight and obese adolescents.

**TABLE 1:** Participant characteristics stratified by group (NSUs vs. PSUs).

Parameter	NSUs (*N* = 809)	PSUs (*N* = 151)	*p*	Effect size
Demographics				
Age (years), M ± SD	14.7 ± 0.5	15.1 ± 0.6	< 0.001	*d* = 0.75
Screen use and PSU				
Screen time (min/day), median [IQR]	155 [105, 215]	435 [380, 505]	< 0.001	*r* = 0.70
SAS‐SV score, M ± SD	25.4 ± 6.2	46.8 ± 7.9	< 0.001	*d* = 3.05
Anthropometrics				
WHO BMI categories, *n* (%)				V = 0.33
Underweight	214 (26.5%)	35 (23.2%)	0.42	
Normal	531 (65.6%)	48 (31.8%)	< 0.001	
Overweight	58 (7.2%)	49 (32.5%)	< 0.001	
Obese	6 (0.7%)	19 (12.6%)	< 0.001	
Physical activity (MET‐min/week), median [IQR]				
Vigorous	480 [120, 1080]	0 [0, 240]	< 0.001	*r* = 0.35
Moderate	0 [0, 120]	0 [0, 0]	< 0.001	*r* = 0.20
Walking	132 [66, 396]	132 [66, 264]	0.54	*r* = 0.03
Other measures				
Rest time (min/day), median [IQR]	40 [25, 60]	90 [60, 150]	< 0.001	*r* = 0.40
Years of sports practice, median [IQR]	0.0 [0.0, 2.0]	0.0 [0.0, 0.0]	< 0.001	*r* = 0.25
FAS categories, *n* (%)				V = 0.45
No significant fatigue	248 (30.7%)	5 (3.3%)	< 0.001	
Mild‐to‐moderate fatigue	514 (63.5%)	70 (46.4%)	< 0.001	
Severe fatigue	47 (5.8%)	76 (50.3%)	< 0.001	

*Note:* Data presented as mean ± SD for normal continuous variables and median [interquartile range] for non‐normal continuous variables. Categorical data as *n* (%). Effect sizes: Cohen's *d* (*t*‐tests), *r* (Mann–Whitney *U*), Cramer's V (chi‐square). A median of zero MET‐min/week indicates that ≥ 50% of participants in the group reported no activity at that intensity. Statistical significance: *p* < 0.05, *p* < 0.01, *p* < 0.001.

Abbreviations: FAS = Fatigue Assessment Scale; IPAQ = International Physical Activity Questionnaire; NSUs = non‐problematic smartphone users; PSUs = problematic smartphone users; SAS‐SV = Smartphone Addiction Scale—Short Version; WHO BMI = World Health Organization body mass index.

Statistically significant differences were also observed in physical activity and fatigue profiles. Adolescents with PSU reported considerably lower engagement in vigorous and moderate physical activity, alongside longer daily rest time, fewer years of sports participation and a substantially higher prevalence of severe fatigue compared with NSUs (all *p* < 0.001).

### 3.2 Sex Differences in Study Variables

Table 2 summarizes participant characteristics stratified by biological sex. Statistically significant sex differences were observed across multiple domains. Males exhibited higher Smartphone Addiction Scale—Short Version scores and longer daily screen time and reported greater engagement in vigorous and moderate physical activity compared with females (all *p* < 0.01). In contrast, females reported higher levels of walking activity and longer daily rest time.

**TABLE 2:** Participant characteristics stratified by sex (female vs. male).

Parameter	Female (*N* = 506)	Male (*N* = 454)	*p*	Effect size
Demographics				
Age (years), M ± SD	14.8 ± 0.5	14.9 ± 0.6	0.002	*d* = 0.20
Screen use and PSU				
Screen time (min/day), Median [IQR]	195 [140, 270]	230 [160, 320]	< 0.001	*r* = 0.15
SAS‐SV score, M ± SD	32.1 ± 7.8	38.2 ± 8.5	< 0.001	*d* = 0.75
Anthropometrics				
WHO BMI categories, *n* (%)				*V* = 0.12
Underweight	142 (28.1%)	106 (23.3%)	0.12	
Normal	316 (62.4%)	263 (58.0%)	0.03	
Overweight	42 (8.3%)	65 (14.3%)	< 0.001	
Obese	6 (1.2%)	20 (4.4%)	< 0.001	
Physical activity (MET‐min/week), median [IQR]				
Vigorous	240 [0, 960]	600 [120, 1440]	< 0.001	*r* = 0.20
Moderate	0 [0, 120]	40 [0, 240]	0.002	*r* = 0.10
Walking	165 [66, 462]	132 [66, 396]	0.03	*r* = 0.07
Other measures				
Rest time (min/day), median [IQR]	50 [30, 80]	45 [30, 70]	0.01	*r* = 0.08
Years of sports practice, median [IQR]	0.0 [0.0, 1.0]	0.0 [0.0, 2.0]	< 0.001	*r* = 0.18
FAS categories, *n* (%)				*V* = 0.15
No significant fatigue	144 (28.5%)	109 (24.0%)	0.02	
Mild‐to‐moderate fatigue	325 (64.2%)	259 (57.1%)	0.01	
Severe fatigue	37 (7.3%)	86 (18.9%)	< 0.001	

*Note:* Data presented as mean ± SD for normal continuous variables (age, SAS‐SV) and median [interquartile range] for non‐normal continuous variables (all others). Categorical data as *n* (%). Effect sizes: Cohen's *d* (*t*‐tests), r (Mann–Whitney *U*), Cramer's V (chi‐square). Statistical significance: *p* < 0.05, *p* < 0.01, *p* < 0.001.

Statistically significant differences were also found in weight status (BMI distribution), with a higher prevalence of overweight and obesity among males. Furthermore, males reported more years of sports practice but showed a higher prevalence of severe fatigue compared with females (all *p* < 0.001).

### 3.3 Correlates of Smartphone Use Across User Groups

Table 3 presents Spearman's correlations between smartphone use indicators and health outcomes. Both SAS‐SV scores and daily screen time were strongly negatively correlated with physical activity, particularly vigorous activity, and strongly positively correlated with fatigue scores (all *p* < 0.001). These associations were consistently stronger within the PSU group. Small but statistically significant positive correlations were observed with BMI z‐scores, whereas sports practice exhibited moderate negative correlations with smartphone use measures (all *p* < 0.05).

**TABLE 3:** Spearman's is correlations (ρ) between smartphone use and health outcomes, stratified by user group.

Outcome	Variable	NSUs (*N* = 809)	PSUs (*N* = 151)
Physical activity			
IPAQ score	SAS‐SV	−0.50	−0.60
	Screen time	−0.48	−0.58
Vigorous activity	SAS‐SV	−0.55	−0.65
	Screen time	−0.52	−0.62
Moderate activity	SAS‐SV	−0.25	−0.35
	Screen time	−0.23	−0.33
Walking	SAS‐SV	−0.05	−0.15
	Screen time	−0.04	−0.12
Other measures			
Rest time	SAS‐SV	0.30	0.40
	Screen time	0.28	0.38
FAS global score	SAS‐SV	0.70	0.80
	Screen time	0.60	0.70
WHO BMI (z‐score)	SAS‐SV	0.18	0.25
	Screen time	0.15	0.20
Years of sports practice	SAS‐SV	−0.38	−0.50
	Screen time	−0.35	−0.45

*Note:* All correlations for vigorous activity, IPAQ score, FAS score and rest time are statistically significant at *p* < 0.001. Correlations for moderate activity and sports practice are statistically significant at *p* < 0.01. Correlations for BMI are statistically significant at *p* < 0.05. Correlations for walking are no statistically significant (*p* > 0.05).

Abbreviation: SAS‐SV = Smartphone Addiction Scale—Short Version.

Tables 4 and 5 present the results from adjusted logistic regression models examining protective and risk factors for PSU by sex. Sports practice was consistently associated with lower odds of adverse outcomes. A statistically significant sex interaction was observed for its protective effect against PSU (*p* = 0.03), indicating a stronger protective association in females than in males (Table 4).

**TABLE 4:** Protective effects of sports practice on health outcomes, stratified by Sex.

Outcome	Sports practice effect	Female (OR, 95% CI)	Male (OR, 95% CI)	Sex interaction (*p*)
PSU risk (SAS‐SV)	OR per year of practice	0.4 (0.3–0.6)	0.6 (0.4–0.9)	0.03
Obesity	OR per year of practice	0.5 (0.3–0.8)	0.5 (0.3–0.8)	0.01
Severe fatigue	OR per year of practice	0.4 (0.3–0.6)	0.4 (0.3–0.6)	0.20

*Note:* Table presents adjusted odds ratios (OR) and 95% confidence intervals (CI) from logistic regression models examining the association between each additional year of sports practice and health outcomes. All models are controlled for age and screen time. The reference category for sports practice was 0 years. Sex interaction *p*‐values were obtained from models including a sports practice × sex interaction term and indicate whether associations differed significantly between females and males.

**TABLE 5:** Adjusted odds ratios for PSU risk factors, stratified by sex.

Category	Female (OR, 95% CI)	Male (OR, 95% CI)	Interaction (*p*)
PSU	1.0 (Reference)	1.3 (0.9–1.8)	0.12
IPAQ low activity	5.2 (3.2–8.4)	6.0 (3.8–9.5)	0.04
FAS severe fatigue	4.0 (2.4–6.7)	4.5 (2.7–7.5)	0.20
WHO obesity	3.8 (2.1–6.9)	5.5 (3.2–9.4)	0.01
Sports practice	0.4 (0.3–0.6)	0.6 (0.4–0.9)	0.03

*Note:* Table presents adjusted odds ratios (OR) and 95% confidence intervals (CI) from logistic regression models examining risk and protective factors for problematic smartphone use (PSU). All models are adjusted for age and BMI. The female group serves as the reference (OR = 1.0). For sex prevalence comparisons, females served as the reference group (OR = 1.0). For all other predictors, the reference category was the absence of the condition (e.g., non‐low physical activity, non‐obese, no severe fatigue or no sports practice). Interaction *p*‐values were derived from models including predictor × sex interaction terms and assess whether associations with PSU differed by sex.

In contrast, low physical activity and obesity were significant risk factors for PSU in both sexes. Statistically significant interactions indicated that these associations were stronger in males (interaction *p* = 0.04 and *p* = 0.01, respectively; Table 5). Severe fatigue was associated with higher PSU risk for both males and females, with no evidence of effect modification by sex (interaction *p* = 0.20).

## 4 Discussion

This study aimed to determine the prevalence of PSU and its associations with health behaviours in a sample of Tunisian adolescents. The main findings indicate a PSU prevalence of 15.7%, with statistically significant associations identified with male sex and mid‐adolescence. PSU was correlated with adverse health outcomes, including higher screen time, lower physical activity, higher BMI and more severe fatigue. Sports participation was associated with a protective effect against these negative outcomes, with this association being stronger for females.

The identified prevalence of 15.7% is lower than global pooled estimates of 37.1% reported by Lu et al. (2024) but represents a considerable public health concern. This figure aligns with regional studies in North Africa (Bouazza et al. 2023; Sfendla et al. 2018), suggesting that PSU is a cross‐border issue within the Maghreb. The association between mid‐adolescence (ages 15–16) and increased PSU is consistent with developmental models that highlight this period as one of heightened social sensitivity and reward‐seeking behaviour (Keating 2024).

Consistent with the displacement hypothesis, PSU was strongly associated with a sedentary lifestyle. Adolescents with PSU reported significantly longer screen time (> 7 h daily) and lower levels of moderate‐to‐vigorous physical activity. This clustering of screen‐based inactivity is particularly concerning in Tunisia, where baseline physical activity among youth is already critically low (Chaabane et al. 2021). Furthermore, over half of the adolescents with PSU reported severe fatigue, which is likely linked to the documented disruptions in sleep quality and circadian rhythms associated with excessive screen use (Jeong et al. 2023). This suggests a potential cyclical relationship where fatigue reduces motivation for physical activity, further reinforcing sedentary behaviours.

A positive association was also observed between PSU and higher BMI. Although the effect size was modest, its relevance is underscored by the study's design, which excluded adolescents with potential eating disorders (SCOFF score ≥ 2) to isolate the relationship between PSU and BMI from confounding dietary pathologies. This finding aligns with meta‐analytic evidence showing that even minor BMI increases during adolescence can be associated with significant long‐term health implications (Mayarestya et al. 2021).

A key finding of this cross‐sectional study is the consistent association between sports participation and positive outcomes. Each additional year of sports practice was linked to lower odds of PSU, severe fatigue and obesity. This relationship may be explained by self‐determination theory (Deci and Ryan 2008), whereby sports provide a healthy avenue for fulfilling psychological needs for competence, autonomy and relatedness, potentially reducing reliance on compensatory smartphone use. Notably, this protective association was significantly stronger for females. In the Tunisian context, where cultural norms may restrict girls' access to public spaces, organized sports may provide a critical avenue for physical activity, social engagement and autonomy (Welhezi et al. 2013), which may explain the stronger protective effect observed.

### 4.1 Practical Implications for Prevention

The present findings have direct implications for adolescent health promotion strategies. Prevention efforts should extend beyond generalized screen‐time warnings and prioritize the provision of structured, attractive alternatives to sedentary smartphone use. School‐based programmes should emphasize sustained engagement in organized physical activity, with particular attention to gender‐responsive approaches that address barriers to participation among adolescent girls. At the family level, guidance should encourage the regulation of daily routines through scheduled offline periods devoted to physical and social activities. In clinical contexts, routine assessment of smartphone use behaviours should be considered when adolescents present with fatigue, obesity or reduced motivation.

### 4.2 Limitations and Future Directions

Several limitations merit consideration. First, the cross‐sectional design limits causal interpretation of the observed associations, underscoring the need for longitudinal and prospective studies. Second, the exclusion of adolescents with neurodevelopmental disorders or physical disabilities, while intended to reduce confounding, restricts the generalizability of the findings to otherwise healthy populations. Future research should incorporate these groups using adapted assessment protocols. Third, although objective measures of screen time were employed, data on the qualitative aspects of smartphone use (e.g., content type and usage context) were unavailable; integrating such information would strengthen future analyses.

## 5 Conclusion

This study identified a 15.7% prevalence of PSU in a sample of Tunisian adolescents, with higher rates associated with mid‐adolescence and male sex. PSU was statistically significantly associated with adverse health indicators, demonstrating a clustering of physical inactivity, elevated screen time, fatigue and higher BMI. A key finding was the protective role of sports participation, which was associated with reduced risk for these negative outcomes. This protective effect was notably stronger against PSU itself in female adolescents. These findings highlight the public health importance of PSU and underscore the potential of physical activity, particularly structured sports, as a focal point for preventive strategies.

## Author Contributions

**Mohamed Yaakoubi:** conceptualization, methodology, formal analysis, writing – original draft. **Ahmed Ghorbel:** investigation, data curation, writing – review and editing, validation. **Hiba Abdelkafi:** investigation, writing – review and editing. **Liwa Masmoudi:** formal analysis, visualization. **Adnene Gharbi:** investigation, writing – original draft, visualization, supervision. **Omar Trabelsi:** investigation, writing – original draft, supervision.

## Funding

The authors have nothing to report.

## Ethics Statement

This study received ethical approval from the local Committee for the Protection of Persons (CPP SUD; Approval No. 0554/2023). The research was conducted in full compliance with the ethical standards outlined in the Declaration of Helsinki 2013 and the guidelines set forth by the British Educational Research Association (BERA, 2018), ensuring the protection, dignity and rights of all participants involved.

## Conflicts of Interest

The authors declare no potential conflicts of interest.

## Data Availability

The data that support the findings of this study are available from the corresponding author upon reasonable request.
